# A new dosing regimen of ropeginterferon alfa-2b is highly effective and tolerable: findings from a phase 2 study in Chinese patients with polycythemia vera

**DOI:** 10.1186/s40164-023-00415-0

**Published:** 2023-06-21

**Authors:** Jie Jin, Lei Zhang, Albert Qin, Daoxiang Wu, Zonghong Shao, Jie Bai, Suning Chen, Minghui Duan, Hu Zhou, Na Xu, Sujiang Zhang, Xuelan Zuo, Xin Du, Li Wang, Pei Li, Xuhan Zhang, Yaning Li, Jingjing Zhang, Wei Wang, Weihong Shen, Oleh Zagrijtschuk, Raymond Urbanski, Toshiaki Sato, Zhijian Xiao

**Affiliations:** 1 Department of Hematology, The First Affiliated Hospital, Zhejiang University School of Medicine, Hangzhou, Zhejiang China; 2 State Key Laboratory of Experimental Hematology, National Clinical Research Center for Blood Diseases, Institute of Hematology and Blood Diseases Hospital, Chinese Academy of Medical Sciences and Peking Union Medical College, Tianjin, 300020 China; 3 Medical Research & Clinical Operations, PharmaEssentia Corporation, Taipei, Taiwan, Republic of China; 4 PharmaEssentia Biotech (Beijing) Limited, Beijing, China; 5 The Second Hospital of Tianjin Medical University, Tianjin, China; 6 The First Affiliated Hospital of Soochow University, Suzhou, Jiangsu China; 7 Peking Union Medical College Hospital, Chinese Academy of Medical Sciences and Peking Union Medical College, Beijing, China; 8 Affiliated Cancer Hospital of Zhengzhou University and Henan Cancer Hospital, Zhengzhou, Henan China; 9 Nanfang Hospital of Southern Medical University, Guangzhou, Guangdong China; 10 Ruijin Hospital, Shanghai Jiaotong University School of Medicine, Shanghai, China; 11 Zhongnan Hospital, Wuhan University, Wuhan, Hubei China; 12 Shenzhen Second People’s Hospital, Shenzhen, Guangdong China; 13 The First Affiliated Hospital of Chongqing Medical University, Chongqing, China; 14 Huashan Hospital of Fudan University, Shanghai, China; 15 Division of Life Sciences and Medicine, The First Affiliated Hospital of USTC, University of Science and Technology of China, Hefei, China; 16 PharmaEssentia USA Corporation, Burlington, MA USA; 17 PharmaEssentia Japan K. K, Motoakasaka, Minato-Ku, Tokyo, Japan; 18 Institute of Hematology and Blood Diseases Hospital, Chinese Academy of Medical Sciences & Peking Union Medical College, Tianjin, China

**Keywords:** Ropeginterferon alfa-2b, New dosing regimen, Chinese patients with polycythemia vera, Complete hematologic response, Molecular response, *JAK2*^V617F^ allele burden

## Abstract

Ropeginterferon alfa-2b represents a new-generation pegylated interferon-based therapy and is administered every 2–4 weeks. It is approved for polycythemia vera (PV) treatment in the United States and Europe with a starting dose of 100 µg (50 µg for patients receiving hydoxyurea) and intra-patient dose titrations up to 500 µg at 50 µg increments, which took approximately 20 or more weeks to reach a plateau dose level. This study aimed to assess ropeginterferon alfa-2b at an alternative dosing regimen with a higher starting dose and quicker intra-patient dose titrations, i.e., the 250–350–500 μg schema, in 49 Chinese patients with PV with resistance or intolerance to hydroxyurea. The primary endpoint of the complete hematologic response rate at treatment weak 24 was 61.2%, which was notably higher than 43.1% at 12 months with the approved dosing schema. The *JAK2*^V617F^ allele burden decreased from baseline to week 24 (17.8% ± 18.0%), with one patient achieving a complete molecular response. Ropeginterferon alfa-2b was well-tolerated and most adverse events (AEs) were mild or moderate. Common AEs included alanine aminotransferase and aspartate aminotransferase increases mostly at grade 1 or 2 levels. Patients did not present with jaundice or significant bilirubin level increase. No grade 4 or 5 AEs occurred. Seven patients (14.3%) experienced reversible, drug-related grade 3 AEs. No AEs led to treatment discontinuation. Ropeginterferon alfa-2b at the 250–350–500 μg regimen is highly effective and well-tolerated and can help patients achieve greater and rapid complete hematologic and molecular responses.

*Clinical Trial Registration: *This trial is registered at ClinicalTrials.gov (Identifier: NCT05485948) and in China (China National Medical Products Administration Registration Number: CTR20211664).

## To the Editor

Patients with Polycythemia vera (PV) are at an increased risk of developing thromboembolic events due to increased blood cell counts and have a long-term risk of neoplastic progression to myelofibrosis or acute myeloid leukemia [1]. Ropeginterferon alfa-2b represents a new-generation pegylated interferon (IFN)-based therapy [2–7]. It has been approved for the treatment of PV in the United States, Europe, and other countries or regions, including Macao, China. Ropeginterferon alfa-2b is used at a starting dose of 100 µg [or 50 µg for patients under hydroxyurea (HU) treatment], with intra-patient dose increments of 50 µg to a maximum recommended dose of 500 µg [8]. It comprises multiple steps and could take up to more than 20 weeks to reach the plateau dose level [8, 9]. This current study aimed to assess whether ropeginterferon alfa-2b treatment at a starting dose of 250 µg, followed by 350 µg two weeks later and then 500 µg from week 4 onwards, i.e., the 250–350–500 µg dosing regimen, could achieve rapid and good clinical efficacy with tolerability within 24 weeks of treatment.

**Fig. 1:**
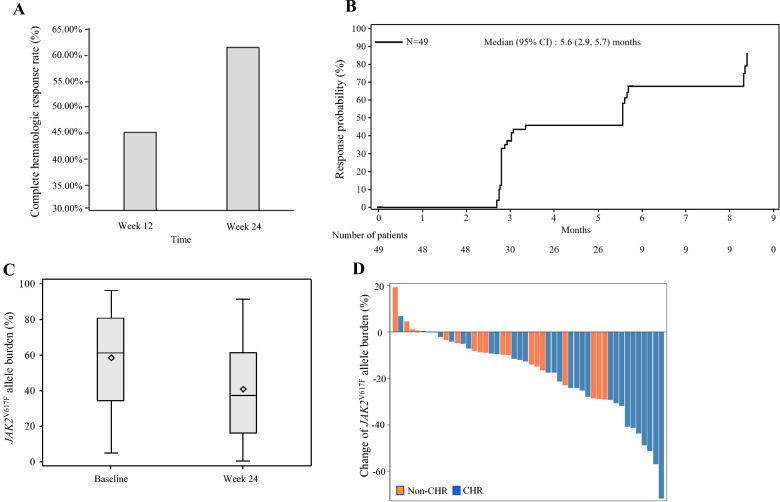
**A** Bar graph showing the complete hematologic response (CHR) rates at 12 and 24 weeks. **B** Kaplan–Meier plot showing time to CHR. **C** Graph showing mean *JAK2*^V617F^ allele burden change. Diamond indicates the mean value. **D** Waterfall plot showing the change of the *JAK2*^V617F^ allele burden in individual patients. Patients who had a CHR are indicated in blue

This study enrolled 49 patients. One patient withdrew consent during the study and 48 patients completed the 24-week treatment according to protocol. The mean patient age at enrolment was 53 years. All patients had previously received HU and were intolerant to HU. More than half of the patients (61.2%) received prior IFN therapy. Patients with prior IFN treatment needed to have a wash-out time of at least 14 days and were negative for ropeginterferon alfa-2b binding antibodies [10]. The primary end point of the study, namely the complete hematologic response (CHR) rate at week 24 without the need for phlebotomy or erythrocyte apheresis in the preceding 3 months, and its 95% confidence interval (CI) was 61.2% [46.2%, 74.8%]. The CHR rate change from baseline to 12 and 24 weeks is shown in Fig. 1A. The CHR rate at week 24 (61.2%) is notably higher than that at 12 months observed in the PROUD-PV study and a Phase II study by Edahiro et al. (43.1% and 51.7%, respectively) [8, 11]. The approved slow titration schema was used in the PROUD-PV study and the Phase II study by Edahiro et al. The median time to CHR was approximately 22.2 weeks or 5.6 months [95% CI: 2.9, 5.7] (Fig. 1B). Mean hematocrit, platelet, and WBC levels decreased over time from baseline to week 24: − 4.7%, − 252.6 × 10^9^/L, and − 5.7 × 10^9^/L, respectively, and improved to be within normal ranges at week 24. The *JAK2*^V617F^ allele burden reduced in 41 out of 48 patients (85.4%). The mean change in the *JAK2*^V617F^ allele burden is shown in Fig. 1C. The *JAK2*^V617F^ allele burden at week 24 was 40.7% ± 27.5%. The mean change from baseline to week 24 was − 17.8%. The change of *JAK2*^V617F^ allele burden in individual patients is shown in Fig. 1D. One patient achieved a complete molecular response. Partial molecular response was observed in 23 patients (46.9%) and 13 (26.5%), based on the 2009 and 2013 criteria, respectively [12]. For patients with at least 20% *JAK2*^V617F^ allele burden at baseline, 13 of the 43 patients (30.2%) achieved partial molecular response, as defined by ≥ 50% reduction from the baseline.

Ropeginterferon alfa-2b was well-tolerated in the study. The targeted optimal dose of 500 μg was reached in all patients except one. Adverse events (AEs), which were mostly mild or moderate, were reported in 48 out of 49 patients. The most common AEs with an incidence ≥ 10% included an increase in alanine aminotransferase (ALT; 25/49, 51.0%) and aspartate aminotransferase (AST; 25/49, 51.0%). Most ALT and AST increases were grade 1 or grade 2, except in one patient who had a grade 3 ALT increase. However, this patient did not have a bilirubin increase or symptoms or signs such as jaundice. There were no grade 4 or 5 AEs. Grade 3 AEs that were possibly drug-related occurred in seven of the 49 patients (14.3%), which were all resolved except one patient who had grade 3 gamma glutamyl transferase increase recovering to the grade 2 level. No bilirubin levels at grade 2 or above were observed. No AEs led to treatment discontinuation. Serious AEs (SAE) that were possibly treatment-related were reported in two patients (4.1%). One patient experienced grade 3 ALT and grade 2 AST increases without elevated bilirubin or clinical symptoms at the dose of 500 μg. The patient was admitted to hospital for further check-up, and therefore reported to have an SAE. The dose was reduced to 350 μg and then to 250 μg, leading to the normalization of the aminotransferases. The dose was then adjusted and maintained at 350 μg. The second case was a 64-year-old patient experiencing an SAE of grade 3 pneumonia at the dose of 500 μg. The patient recovered before the next visit without any action taken on the dose. The common AEs occurring in ≥ 10% of patients are summarized in Table 1.

**Table 1:** Treatment-emergent adverse events (TEAEs) occurring in ≥ 10% of patients

System organ class Preferred term	Patients (n = 49)^a^					
	Grade 1	Grade 2	Grade 3^b^	Grade 4	Grade 5	Total
	n (%)	n (%)	n (%)	n (%)	n (%)	n (%)
Metabolism and nutrition disorders						
Hyperuricemia	16 (32.7%)	0	0	0	0	16 (32.7%)
Hypertriglyceridemia	9 (18.4%)	0	2 (4.1%)	0	0	11 (22.4%)
Decreased appetite	5 (10.2%)	0	0	0	0	5 (10.2%)
General disorders and administration site conditions						
Fatigue	7 (14.3%)	3 (6.1%)	0	0	0	10 (20.4%)
Skin and subcutaneous tissue disorders						
Alopecia	9 (18.4%)	0	0	0	0	9 (18.4%)
Infections and infestations						
Urinary tract infection	5 (10.2%)	4 (8.2%)	0	0	0	9 (18.4%)
Musculoskeletal and connective tissue disorders						
Back pain	5 (10.2%)	0	0	0	0	5 (10.2%)
Renal and urinary disorders						
Albuminuria	6 (12.2%)	1 (2.0%)	0	0	0	7 (14.3%)
Nervous system disorders						
Hypoesthesia	6 (12.2%)	0	0	0	0	6 (12.2%)
Investigations						
Alanine aminotransferase increased*	21 (42.9%)	3 (6.1%)	1 (2.0%)	0	0	25 (51.0%)
Aspartate aminotransferase increased**	20 (40.8%)	5(10.2%)	0	0	0	25 (51.0%)
Gamma glutamyl transferase increased***	13 (26.5%)	3 (6.1%)	2 (4.1%)	0	0	18 (36.7%)
White blood cell count decreased^#^	8 (16.3%)	9 (18.4%)	2 (4.1%)	0	0	19 (38.8%)
Neutrophil count decreased	7 (14.3%)	6 (12.2%)	2 (4.1%)	0	0	15 (30.6%)
Lymphocytopenia	2 (4.1%)	6 (12.2%)	1 (2.0%)	0	0	9 (18.4%)
Beta 2 microglobulin urine increased	8 (16.3%)	0	0	0	0	8 (16.3%)
Thrombocytopenia	8 (16.3%)	0	0	0	0	8 (16.3%)
Weight loss	6 (12.2%)	1 (2.0%)	0	0	0	7 (14.3%)
Bilirubin increased^##^	6 (12.2%)	0	0	0	0	6 (12.2%)
Blood alkaline phosphatase increased	6 (12.2%)	0	0	0	0	6 (12.2%)
White blood cell urine positive	5 (10.2%)	0	0	0	0	5 (10.2%)
Lactate dehydrogenase increased	5 (10.2%)	0	0	0	0	5 (10.2%)
Anemia	3 (6.1%)	2 (4.1%)	0	0	0	5 (10.2%)

^a^Patients had previously received hydroxyurea treatment and 61.2% of the patients received prior interferon therapy

^b^In this table representing TEAEs occurring in ≥ 10% of patients, ten grade 3 TEAEs occurred in eight patients. Among them, possibly treatment-related TEAEs occurred in five patients

^*^Four patients (8.2%) had a prior history of grade 1 alanine aminotransferase increase

^**^One patient (2.0%) had a prior history of grade 1 aspartate aminotransferase increase

^***^Three patients (6.1%) had a prior history of grade 1 gamma glutamyl transferase increase

^#^Two patients received G-CSF management of the blood count decrease during treatment

^##^Two patients (4.1%) had a prior history of grade 1 bilirubin increase

In conclusion, ropeginterferon alfa-2b administered at the 250–350–500 µg dosing regimen shows tolerability, safety, efficacy and molecular response in Chinese patients with PV. The results indicate that ropeginterferon alfa-2b at the 250–350–500 µg dosing regimen is highly effective and well-tolerated. The data provides a treatment option for helping ensure optimal clinical treatment and care of patients with PV. Long-term treatment and follow-up are planned to assess patient progression-free and overall survival.

## Data Availability

The data will be available to external researchers upon reasonable request to PharmaEssentia after ropeginterferon alfa-2b has acquired marketing approval for polycythemia vera treatment in China.
